# Conjugating Existing Clinical Drugs With Gold Nanoparticles for Better Treatment of Heart Diseases

**DOI:** 10.3389/fphys.2018.00642

**Published:** 2018-05-29

**Authors:** Jingwen Zhang, Aiqun Ma, Lijun Shang

**Affiliations:** ^1^Department of Cardiovascular Medicine, First Affiliated Hospital of Xi’an Jiaotong University, Xi’an, China; ^2^School of Chemistry and Biosciences, University of Bradford, Bradford, United Kingdom

**Keywords:** nanoparticles, gold nanoparticles, heart diseases, drugs, animal models

## Abstract

Developing new methods to treat heart diseases is always a focus for basic research and clinical applications. Existing drugs have strong side-effects and also require lifetime administration for patients. Recent attempts of using nanoparticles (NPs) in treating atherosclerosis in animals and some heart diseases such as heart failure and endocarditis have provided hopes for better drug delivery and reducing of drug side-effects. In this mini-review, we summarize the present applications of using gold nanoparticles (GNPs) as a new drug delivery system in diseased hearts and of the assessment of toxicity in using GNPs. We suggest that conjugating existing clinical drugs with GNPs is a favorable choice to provide “new and double-enhanced” potentiality to those existing drugs in treating heart diseases. Other applications of using NPs in the treatment of heart diseases including using drugs in nano-form and coating drugs with a surface of relevant NP are also discussed.

## Introduction

Heart diseases such as heart failure (HF) cause millions of deaths each year (Yancy et al., 2013). Although existing drugs can be used as the first step to delay the development of symptomatic HF, unfortunately the treatment normally cannot effectively control the process of developing HF. Once symptoms develop, patients will have to take drugs for a lifetime in order to improve functional status as well as prognosis, but strong side-effects of these drugs are a remaining concern. Although cardiac transplantation is the most accepted surgical intervention option for end-stage HF, this strategy is tremendously limited. Therefore new treatments are desperately needed.

Nanoparticles (NPs) have been successfully used for both *in vivo* and *in vitro* biomedical research and applications such as drug manufacture, imaging, biological tagging, anticancer drugs, drug delivery, antibiotic development, gene transmission, tissue engineering, etc. Gold nanoparticles (GNPs) especially have been widely used because they are easy to make and stable. In addition, the gold element itself possesses qualities suitable in a biological-medical area such as being bioinert, having low toxicity, and low immunogenicity. Recent studies have gained positive results using NPs treating cancer in clinical trials. For example, pegylated liposomal doxorubicin HCl (CAELYX/Doxil) was developed and used in metastatic breast cancer phase III clinical trials. It proved to reduce cardiotoxicity and to have comparable efficacy to conventional doxorubicin (O’Brien et al., 2004). In skin cancer cells, GNPs help to deliver drugs to target telomerase, directly halting the growth of the cancer (NCRI, 2016). These accurate deliveries of NPs in cancer treatment suggest that GNPs may be helpful to deliver drugs to heart muscle cells, even to target mitochondria without much toxicity.

In this mini-review, we summarize the present applications of using GNPs as a new drug delivery system in the treatment of heart diseases. We firstly summarize studies in the distribution of GNPs in both normal and diseased hearts, and then assess the toxicity of using GNPs. We further discussed ways of improving the efficiency of delivery and suggest that conjugating existing clinical drugs with GNPs would give “new and double-enhanced” potential in treating heart diseases, as this makes drug delivery more efficient and accurate into the heart muscle cells. Other applications of using NPs in the treatment of heart diseases, including using drugs in nano-form and coating drugs with a surface of relevant NP are also identified.

## Size-Dependent Accumulation of Gnps in Normal Hearts

Better understanding of the distribution of GNPs after administration in the body especially in the heart is essential to understand its effects on the heart. Studies have found that the distribution of GNPs in the heart tissue of normal rats and mice by single intravenous injection is size-dependent (De Jong et al., 2008; Sonavane et al., 2008). For a short-term of 24 h after injection, GNPs with a diameter between 10 and 250 nm were detected in various organs including heart, whereas the larger particles were only found in blood, liver and spleen. For a long-term study of 7 days and several months after injection, inductively coupled plasma-mass spectrometry (ICP-MS) detection showed that small GNPs (13 nm) accumulated in higher levels in the heart compared with GNPs of 4 and 100 nm. The peak level for these small GNPs (13 nm) occurred at 1 month in the heart, and remained high at different time points including 30 min, 4 h, 24 h, 7 days, and 1 month; and then dropped to half at 3 months and almost eliminated at 6 month. This trend is the same with GNPs of 4 nm, but a reversed trend was observed for those of 100 nm. The peak of GNPs size of 100 nm occurred at 6 months. Although small GNPs of 13 nm have favorable accumulation in the heart compared with other sizes, the whole accumulation of GNPs in the heart is only one seventh of the amount in the liver, which ranked as the second high accumulation organ, and one forty-fifth of the amount in the spleen, which ranked as the first (Cho et al., 2010). By intra-oesophageal instillation, ultrasmall GNPs (1.4 nm) were mostly found in the highest accumulation in secondary organs. When considering the accumulation in the heart, GNPs of 18 nm showed a higher accumulation compared to other sized particles (Schleh et al., 2012). In conclusion, small GNPs around 13∼18 nm can easily accumulate in the heart.

## Size-Dependent Accumulation of Gnps in Diseased Hearts

Although it has been accepted that small size GNPs can easily accumulate in normal heart tissue by different methods of administration, tissue distribution of GNPs in a diseased heart can be different. A recent study found that GNPs can be enriched more in ischemic heart tissue and HF than in a normal control. Lundy et al. (2016) found that the optimal sizes of NPs for passive targeting of the injured left ventricle immediately following cardiac ischaemia-reperfusion injury in mice was between 20 and 200 nm. Spivak et al. (2013) found that GNPs of size 30 nm have higher cardio-protective effects in doxorubicin-induced HF rats than that induced by Simdax alone. From the aforementioned experiments, diseased hearts demonstrate that larger sizes of NPs are accumulated optimally. However, larger GNPs more than 200 nm cannot be used in diseased hearts. Another study (Lundy et al., 2016) showed that GNPs of 500 nm and larger also have significantly increased retention in the heart following I/R injury, but they were shown to be much greater off-target retention in the spleen. Larger NPs (1 and 2 μm) inside blood vessels mainly appeared as clusters which may be physically entrapped within small capillaries of the heart. In conclusion, larger GNPs can accumulate in diseased hearts better than in normal hearts and the process is also size-dependent as in normal hearts. Larger sized GNPs can target the diseased hearts but will become entrapped within small capillaries.

## Toxicity Assessment of Gnps to the Heart

Toxicity of GNPs to the heart has to be fully understood before using GNPs in the treatment of heart diseases. It is commonly accepted that no observable toxicity occurs in the kidney or liver despite the accumulation of gold in these tissues after repeated administration of GNPs, but whether it will cause toxicity to the cardiovascular system has not been determined (Rambanapasi et al., 2016). The length and the mode of GNPs administration, and particle size are the main factors influencing the toxicity of GNPs to heart *in vivo* (Xia et al., 2016). Toxicity of GNPs to the heart can also be reviewed indirectly by evaluating oxidative stress and alterations in energy metabolism in animal models. The results are summarized as below.

### The Lengths of Administration

For a sub-acute cardiac toxicity assessment, Yang et al. (2013) used an isoproterenol (ISO) induced cardiac remodeling model to investigate the cardiac bio-distribution of polyethylene glycol (PEG)-coated GNPs (13 nm) and their effects on cardiac function, structure and inflammation where GNPs were injected intravenously for seven consecutive days. Their results showed that under normal physiological conditions the increased accumulation of GNPs in the heart had no effect on the function of the heart. Neither did they aggravate ISO-induced cardiac hypertrophy, cardiac fibrosis, or cardiac inflammation. For chronic cardiac toxicity evaluation of GNPs in normal mice, repeated administration of 10 nm PEG-GNPs via tail veins for 14 consecutive days, the LVIDd (left ventricular end-diastolicinner-dimension), LVMass (left ventricular mass), and HW/BW (heart weight/body weight) were significantly increased; but there are no changes for 4 or 12 weeks. These results indicated that the accumulation of small size GNPs can induce cardiac hypertrophy in normal mice, but this effect can be reversed during the washout period (Yang et al., 2016). Although these two studies were both using similar size of small NPs, the administration length were different and these produced contradictory results regarding cardiovascular toxicity. The possible reason for this is that the longer injection period than 7 days may lead to reversible toxicity.

### The Modes of Administration

(Abdelhalim, 2011a,b) studied the toxicity of GNPs on heart tissue in normal rats through intraperitoneal administration. They discovered that rats treated with GNPs 50 or 100 μl of 10 and 20 nm sizes for 3 or 7 days demonstrated congested heart muscle with prominent dilated blood vessels, scattered and extravasations of red blood cells, focus of muscle hyalinosis, disturbed muscle fascicles, dense prominent focus of inflammatory cells infiltrated by small lymphocytes, and few plasma cells, while rats treated with GNPs 50 or 100 μl of 50 nm size for 3 or 7 days demonstrated normal looking heart muscle with normal muscle direction and fascicles, and very few scattered small lymphocytes (Abdelhalim, 2011a,b). The different levels of toxicity induced by intraperitoneal administration of GNPs were size-dependent, with smaller NPs inducing greater toxicity to the heart (Abdelhalim, 2011a,b).

Unlike other cardiovascular disease, HF is mainly developed by consistent vascular stress, and then eventually leads to heart muscle cells injury as well as fibrosis proliferation. In this case, limited *in vivo* studies showed that intravenous injection with GNPs is the most promising way for the treatment as GNPs can then circulate in the vascular system directly without being cleared during digestion process. Nonetheless, various other injection modes have also been applied in coronary heart disease (CHD) using GNPs. CHD often has a fixed injury spot visible on the heart surface or can be located by ECG presentation, which made injection of GNPs to the particular point possible.

### Measuring Oxidative Stress and Energy Metabolism Parameters in Serum

Ferreira et al. (2015) showed that GNPs of 10 and 30 nm sizes can produce oxidative damage after acute and long-term administration, i.e., for a single intraperitoneal injection or repeated injections (once daily for 28 days). These were evaluated by the parameters of oxidative stress and energy metabolism. Results showed that small size GNPs can cause oxidative damage regardless of the length of the administration. The reason for this kind of toxicity is due to DNA damage and alterations in energy metabolism. This result indicated that prolonged administration of small size NPs, e.g., over several years, may also lead to heart toxicity.

### Toxicity of Ultra-Small GNPs

One study (Pan et al., 2013) found that exposure to 50 and 100 μM of Au 1.4MS can cause a string-like heart and other malformations in developing zebrafish embryos. In a patch-clamp experiment (Leifert et al., 2013), 1.4 nm GNPs failed electrophysiology-based safety tests using human embryonic kidney cell line 293 cells expressing human ether-a-go-go-Related gene (hERG). Although ultra-small GNPs can block hERG channels *in vitro*, the high dose of 50 mg/kg *in vivo* did not block the channel and give a trace of heart arrhythmia (Leifert et al., 2013). Due to the fact that NPs can form a protein corona when confronted with protein-containing solutions like serum or full blood (Casals et al., 2010; Lacerda et al., 2010), it is suspected that this process will lead to the enlargement of the diameter of NPs, making it impossible to block hERG channels *in vivo*. Therefore the toxicity of NPs discovered *in vitro* needs to be clarified before applying *in vivo*. The bio-distribution of ultra-small NP in the heart and its toxicity assessment between *in vivo* and *in vitro* remains to be determined.

In conclusion, the toxicity of GNPs to the heart have been examined by using well-accepted techniques including ICP-MS, whole-cell membrane patch-clamp, serum parameter measurement, etc. Although some results are contradictory, the most relevant factors requiring serious consideration regarding its toxicity are the mode of administration, the length of administration and the size of NPs. Long-term exposure to NPs and more *in vivo* studies are required before any conclusions can be made.

## Gnps Conjugated With Clinical Approved Drugs in the Treatment of Heart Diseases

The present drugs used in the treatment of heart diseases are not efficient enough and they also have strong side-effects when patients are required to take the drugs for the duration of their lives in order to control the symptoms and make better prognosis. Making good use of existing drugs and combining them with GNPs to improve the efficiency of delivery is therefore an obvious straight forward and a favorable choice. It would be expected that this conjugation will give “new and double-enhanced” potentiality to existing drugs as drug delivery into the heart muscle cells becomes more efficient and accurate in the conjugation form.

There is only one study using clinical confirmed drugs conjugated with NPs in the treatment of heart disease. Spivak et al. (2013) found that GNPs-Simdax and GNPs (30 nm) both made significant cardio-protective effects in rats with doxorubicin-induced HF, which is higher than that of Simdax treatment alone. Moreover, it seems that the effect of GNPs-Simdax (*P* < 0.01) is better than GNPs (30 nm; *P* < 0.05) only, though there is no difference for rat recovery between GNPs-Simdax and GNPs (30 nm) to treat HF with GNPs in that study. It is probably due to the lack of preferential targeting of GNPs-Simdax than GNPs (30 nm) to target heart tissue.

β-Blockers are another good candidate for this purpose of making NPs conjugation, even better than Simdax, as it can counteract many harmful effects of the hyperactivity of the sympathetic nervous system underlying the volume-overloaded HF (Ni et al., 2013). Metoprolol is a widely used β-blocker, we suspect that when Metoprolol is conjugated with GNPs, it may accumulate much more easily and quickly in heart tissue then Simdax does, because Metoprolol can specifically target β1 receptors which are mostly expressed in the heart. With GNPs targeting heart tissue in HF more easily, GNPs conjugated Metoprolol may show a double efficiency. We are planning to use the widely used β-blocker (Metoprolol) conjugated with GNPs in treating volume-overloaded HF in rats. This might improve potential clinical practice, as increasing the dose of GNPs conjugated Metoprolol is less likely to cause side-effects on other organs.

## Other Applications of Gnps in the Treatment of Heart Diseases

The large surface area-to-volume ratio of GNPs enables their surface to be coated with hundreds of molecules (including therapeutics, targeting agents, and antifouling polymers) (Ghosh et al., 2008). NPs carrying cell growth factors would be useful to amend the damaged heart tissue in coronary arterially disease (Nguyen et al., 2015) as it is difficult to target the diseased area of the heart using traditional administration methods intravenously and it is also dangerous to administer drugs directly into the heart.

Other forms of NPs used in cancer treatment, such as using drugs in nano-form directly and the drug coated with purpose selected GNPs should also be considered in the treatment of the heart diseases both *in vitro* and *in vivo*.

## Conclusion

In this mini-review, we conclude that GNPs show a size-dependent accumulation in both healthy and diseased hearts. Using GNPs conjugated with clinically approved drugs can have positive effects or enhance the efficiency of the “existing clinical” drugs in the treatment of the heart diseases. This effect is possibly due to the fact that GNPs enable accurate targeting and delivery of the drugs to the targeted tissues. This ability may be even more enhanced when GNPs conjugated with “existing clinical” drugs if there is a specific receptor for the drugs existing in the heart. At the same time, the toxicity of GNPs to the heart is still not clear due to the contradictory results in some animal studies.

Gold nanoparticles in the treatment of heart diseases has been gradually proved to be one of the most promising strategies due to its accurate delivery ability to the target diseased tissue and the advantageous ability of carrying drugs with it because of its large surface area-to-volume ratio (Ghosh et al., 2008). As GNPs can form a protein corona when confronted with protein-containing solutions such as serum or full blood (Casals et al., 2010; Lacerda et al., 2010), this can make the diameter and properties of GNPs different *in vivo* from *in vitro*. The lack of animal studies also brings contradictory results. Furthermore, whether GNPs conjugated with drugs exert a better effect than GNPs alone in the treatment of heart disease is still not very clear. Therefore the mechanism for this potential double-enhanced delivery of existing drugs still needs to be further verified. Many puzzles need to be solved before the application of GNPs in the treatment of heart disease can go into clinical trials. Therefore, we propose that a wide range of studies using drugs conjugated with GNPs should be carried out in a variety of heart diseases animal models *in vivo*.

## Author Contributions

JZ, AM, and LS wrote the review.

## Conflict of Interest Statement

The authors declare that the research was conducted in the absence of any commercial or financial relationships that could be construed as a potential conflict of interest.
